# Progress in Surgery

**Published:** 1900-03-17

**Authors:** 


### Progress in Surgery.

### SURGERY OF THE INTESTINES.

**Traumatic Jejunal Fistula** is a rare condition. Cushing[1] relates the case of a man, aged 28, who ten years previously had received a razor wound in the abdomen, which completely severed the intestine in one place and opened it in two others. An intestinal fistula developed, and great emaciation ensued. From the fistula, through which a rosette of mucous membrane usually protruded, issued jets of an acid fluid frequently bile-stained. Attempts to feed through the fistula were unsuccessful. The fistula was closed by a resection of the jejunum, and end-to-end suture over a Halsted's inflated rubber cylinder. Convalescence was uninterrupted, and the patient's weight increased in two months from 93 pounds to 178½ pounds.

**"Anæmia Necrosis" of the Intestine** may, according to Kocher,[2] attend operations on the gastro-intestinal canal. The necrosis of the mucous membrane is produced by a temporary but sufficiently long interruption to the circulation in the mesenteric vessels caused by traction and manipulation during an operation. The occurrence of this anæmia necrosis may account for the rise of temperature, and for the diarrhœa which may occur after operations on the gastro-intestinal canal. Kocher, therefore, makes the following suggestions: First, to make the opening in the abdominal wall large enough to avoid pressure on the vessels of the organs which are pulled out for examination or operation; secondly, to manipulate the mesenteric vessels as little as possible; and lastly, in isolating the field of operation from the rest of the abdomen, not to apply too great a pressure with gauze pads, &c.

**Intestinal Exclusion.**—Le Denton[3] publishes a summary of this operation, which consists in separating a loop of gut by complete division above and below, closing its two cut ends, and uniting (by end to end union best) the cut ends of the intestine above and below the divided loop. The special feature in intestinal exclusion is the next step, the treatment of the excluded coil. Von Eiselsberg and others suture both ends of that coil to the skin. This method allows of the free escape of any morbid material in the loop. Some operators sew only the distal or the proximal end of the loop to the external wound, the other end being closed and sunk in the abdominal cavity. The operation is usually performed on the large intestine, but has also been employed in the small gut. Intestinal exclusion is not suited for cases of acute obstruction. It may answer well in certain cases of tumour of the intestine, in damage to intestine from inflammatory changes, and, above all, in pyo-stercoral and neoplastic fistulæ.

**Duodenal Perforation.**—A case of successful operation for this condition is reported by Johnson,[4] who cites the following statistics: Of 39 cases of duodenal ulcer on record recovery had occurred in 8, in 20 the ulcer was not recognised; in two general peritonitis was thought to exist, in 13 appendicitis, and in three intestinal obstruction. Of 19 cases in which the ulcer was found, though not diagnosticated beforehand, recovery occurred in six.

**Tumours of the Mesentery.**—According to Begonin,[5] operation is indicated in both solid and cystic tumours, as their prognosis, if untreated, is bad. This is due to their growth, and to the peritonitis, cachexia, or intestinal obstruction which they may cause. The author reserves treatment by puncture for blood cysts and unilocular serous cysts, which can be brought into contact with the abdominal wall. He condemns this treatment for all other fluid tumours—echinococcus cysts, dermoid tumours, chylous, and other cystic tumours. In cases in which simple puncture is not indicated, and in cases where it has been tried and failed, incision, if possible, at one operation, or extirpation is to be employed. Extirpation is not indicated in cysts having extensive adhesions. As regards solid tumours of the mesentery, 29 of a series of 36 were treated by extirpation. Fifteen of these died and 14 recovered, making a mortality of 52 per cent. Attention is called to the danger of injuring large vessels, the solar plexus, the ureter, and the bowel. Incisions parallel to the mesenteric vessels are advised, in order to spare them as much as possible, and thus avoid malnutrition of the intestinal wall.

**Intussusception.**—Collier[6] objects to the treatment of intussusception, after injection has failed, by laparotomy and reduction in the way ordinarily described, because of the fatal shock accompanying much exposure and manipulation of the intestines after a free incision. He accordingly recommends a combination of the two methods of injection and manipulation. Under an anæsthetic injection is tried, and the pressure kept up as long as it has any effect upon the tumour. The irrigator is then lowered, and an incision, just large enough to admit a finger, is made over the remnant of the intussesception. The finger introduced, the water pressure is again raised, and the reduction completed by gentle manipulation. Three successful cases are reported. Munro[7] has collected 72 cases of operations for intussusception in the adult. There were 21 recoveries after reduction, and of these 13 were in the ileo-cæcal or colic region. Of 13 fatal reductions, nine were ileo-colic, shock and peritonitis being the main causes of fatal issue. Of 17 recoveries after resection 14 were ileo-colic. Most of them were chronic in character. Of 21 fatal resections nine were ileo-colic seven iliac, one colic, two jejunal, and two unknown—most of them being acute in character.

[1] Brit. Med. Jour., Nov. 4, 1899, Epitome No. 351; and Johns Hopkins Hosp. Bull., July, 1899. [2] Brit. Med. Jour., Nov. 11, 1899, Epitome No. 371; and Deut. Med. Woch., Sept. 14, 1899. [3] Brit. Med. Jour., Nov. 11, 1899, Epitome No. 373; and Rev. de Gynéc. et de Chir. Abdom., Jan.-Feb., 1899. [4] Med. Rec., Dec. 16, 1899, p. 898; and Ann. of Surg., Nov. 1899, p. 634. [5] Ann. of Surg., Dec. 1899, p. 754. [6] Med. Chron., Oct., 1899; and Lancet, Aug. 26, 1899. [7] Med. Rec., Oct. 28, 1899.

